# The Diagnosis of Scarlet Fever

**Published:** 1914-03

**Authors:** W. N. Adkins

**Affiliations:** Atlanta, Ga.


### THE DIAGNOSIS OF SCARLET FEVER.

By W. N. ADKINS, M. D.
Atlanta, Ga.

Of all the acute infectious exanthemata, scarlet fever presents the greatest variety of symptoms which lead to difficulty in diagnosis; not only because this disease presents so great a variety of clinical pictures, but so many other conditions closely resemble one or more of these pictures. Typical cases of scarlet fever are comparatively easy to diagnose; but there are undoubted cases which would tax the skill of the most experienced diagnosticians.

Along about, or before the fifteenth century, both measles and scarlet fever were regarded and written about, as varieties of small pox. Measles was later recognized as a separate disease, and scarlet fever was then regarded as a form of measles, and the first to recognize, and intelligently describe scarlet fever, was Sydenham, during an epidenic in London, in the seventeenth century. Scarlet fever is not strictly a disease of childhood, though the greater majority of cases occur in children. The limit seems to be one year of age. I have never seen a case younger.

The rash of this disease is most variable. It may be intense or faint; universal in its distribution, or only local; however the latter is the exception to the rule. It may be rough in appearance and to the touch; or the reverse; it may be mottled or uniform; it may persist for as much as fifteen days, or fade in three; and again it may be absent altogether; which condition, I admit, is extremely rare. Almost the same variety of possibilities can be said of all the ordinary symptoms. The prodromals may be so slight, as to be considered absent subjectively, or they may be intense and typical in appearance and duration. In short, no single symptom, no matter how pronounced, should be our only basis for a diagnosis; but best consider the symptoms as they appear, and note their duration, before finally deciding on a diagnosis.

I feel safe in making the statement, (though I expect contradiction) that scarlet fever does occur with practically no rise in temperature after the first few hours. Also that no case occurs without sore throat, and either nausea or vomiting. The throat symptoms are present, even if not subjective. The hyperemia is always objectively appreciable on the fauces. The vomiting may occur without accompanying nausea, or the nausea without the vomiting. Another prodromal symptom I have always found, is some degrees of cervical adenitis; be it ever so slight. There is usually an enlargement of the glands in the groin; which however, may not occur until the eruption has made its appearance. You can usually foretell the severity of the infection, by the extent of the prodromals, and particularly the throat and glandular involvement, which always increases with the increasing toxemia. If the rash is to be intense, it is so from the beginning, and if to be a hemorrhagic type, this begins early; the same being true of mottling and other irregularities. The intense rash of scarlet fever is unique, and in the locations where it is heaviest, will be seen white papillae rising clear and distinct, above the surface of the underlying rash.

I have often heard the strawberry tongue of scarlet fever, given as an example of a pathognomonic symptom. Of the various symptoms, I have found the tongue one of the most unreliable; never having seen the true strawberry tongue appear until the rash was well out, and have fine examples of that type of tongue present in undoubted cases of straight measles. The usual tongue of scarlet fever is coated and furry at the onset, with papillae projecting through this whitish coating. This coating peels from the edges toward the center, beginning first at the tip; and is seldom clean before the end of the first week, at which time the typical strawberry tongue is best seen. This condition exists for variable lengths of time; usually for about ten days or two weeks; gradually fading until the normal state is reached. However, I have seen it exist for a month or more. The appearance of the strawberry tongue will at times be the means of gratifying evidence in confirming a diagnosis.

The exanthem of scarlet fever is the most variable of the cardinal symptoms, but that has its limit. I will make another statement, contradictory to some authorities: Regardless of the degree of intensity of the eruption of true scarlet fever, it never at any time makes its appearance on the face; and if there be an eruption there, I believe it to be due to a co-existing skin lesion, and not to the true exanthem of the disease. Regardless of the intensity of the rash elsewhere, the face at times gives no indication of illness, though there is usually a flush, and the eyelids may be swollen; but the skin is smooth. With this flushed condition, there is a distinct pallor around the mouth, including the chin; this, in my opinion is an important sign of scarlet fever.

Regardless of the intensity or faintness of the rash, it never presents a distinct outline, or margin, between the rash and the normal skin, which is found in some skin diseases, drug and serum rashes; and the rash of scarlet fever never begins to desquamate on the day of its appearance; though heavy rashes will at begin to desquamate before fading. No matter how mild or intense the eruption, or manner of its distribution, it is most marked where surfaces of skin are in contact; at the bend of the elbow and knee; in the axilla and groin. With malignant or hemorrhage small pox, the patient rarely lives more than twenty-four or thirty hours after the eruption has appeared; but the same is not true of scarlet fever, for those cases do not terminate fatally, as early as the second, and rarely the third day. Of the cases of hemorrhagic small pox I have seen, the first appearance of the eruption was markedly like scarlet fever, even the appearance of the throat. This similarity, however, only lasted a short time; turning almost black within twelve hours. The initial eruption of ordinary small pox, does not resemble scarlet fever, but frequently closely resembles measeles.

The pulse and temperature cannot always be relied upon in the diagnosis of scarlet fever, for their height is not always in accordance with the intensity of the rash, or the virulence of the infection, though in malignant cases they are usually high to the end. The pulse and temperature in scarlet fever, follow each other with a marked degree of regularity, even with the complications. The same is not true of diphtheria, for instance, for there you may have a high temperature, with a low irregular pulse, indicating a myocardial complication.

The most difficult stage in which to make a diagnosis in scarlet fever, is the interval between the fading of the eruption, and the beginning of the disquamation. This difficulty is added to when it is not possible to get a history of the preceding symptoms; and so many cases go a week or two in that interval before disquamating. There is only one objective sign that I have ever seen, upon which you can place any dependence; and that is the appearance of the palms of the hands and the soles of the feet. These usually present a hardened, dry appearanc, with a whitish cast, as if some white paint or powder had been rubbed over them, and the lines are quite distinct. This appearance, however, seems to depend upon the severity of the preceding eruption. In looking for this symptom, you must also take into consideration, the occupation of the patient, if he be an adult.

Of the prodromals, the nausea and vomiting probably indicates the severity of some lesion in the stomach, intestines, or kidneys; and when these symptoms are pronounced, and followed by severe throat and grandular involvement, you can feel reasonably sure that the temperature will be high, the pulse rapid, and the rash intense.

Desquamation in scarlet fever, when typical, is familiar to us all; but even though less so than the eruption, it is also variable. The pronounced eruption does not necessarily indicate that a profuse desquamation is to follow. I have seen intense rashes followed by mild desquamation, and only a mild one followed by hardly any peeling at all. I think this depends largely upon the character of the epidermis of the individual. The character of the scales also vary, and they are sometimes so small and light, and the desquamation so mild as to be hardly noticeable, especially if the patient has had frequent baths. Though I have seen measles desquamate on the palms, I have never seen it on the soles. Scarlet fever will usually desquamate on both, and occasionally on neither. The palms and soles are usually flushed, while the rash is out, but like the face, the punctate character of the eruption never appears there. I have seen a few cases desquamate a second time, but only mildly, and whether this was directly due to the disease, I am unable to say. Naturally after desquamation, the skin is very tender, and easily irritated; this may account for a second desquamation. Malnutrition and continued fever will cause a condition very similar to the desquamation of scarlet fever; hence the mere fact that a patient has had a rash, and shows this type of desquamation, does not necessarily mean that he has had scarlet fever. But a case, of that type, with a history of nausea, vomiting and fever, followed by an eruption; should be isolated and treated as scarlet fever. After the rash of measles fades, it leaves pigmented spots upon the skin; which are characteristic. Unfortunately scarlet fever leaves no such evidence.

The time taken for the rash of scarlet fever to become general, after its first appearance, is variable. Ordinarily it takes two or three days; but there is no rule. A skin that is to all appearances normal, may, when seen again in three hours, show an intense scarlet fever eruption. But the same is not true in the length of time it will take to fade. No matter how mild the eruption, or how suddenly it appears; once it is out, it will be there for at least three days, with practically no change in its character; unless it becomes hemorrhagic. This is not true of most drug and serum rashes. When pressure is exerted upon the surface of the abdomen, with the distended fingers; a scarlet fever rash will fade, slowly regain its color, and slowly fade again, and these finger marks will be visible sometimes for several hours. Drawing the finger nail sharply across, will leave a distinct line, slightly elevated, with a narrow pale line on either side. These lines will sometimes remain for days. I know of no other rash in which this is true.

The throat of scarlet fever, at times, closely resembles diphtheria; in that in severe infections in both, there is considerable cervical glandular involvement; the throat and nose are filled with mucus, and the patient breathes with difficulty. In the scarlet fever throat, the entire fauces is a fiery red, which is not the case with diphtheria, for there the palate is pale, and the redness is limited to the pharynx, or around the margin of the visible membrane. Another similarity between the throats of these two diseases, is the appearance of the accompanying membrane at times. But the membrane of scarlet fever is white, and due to streptococcus or staphlococcus; while in diphtheria it may be white, gray, greenish, or almost black. Therefore, if the membrane in scarlet fever is of any other color than white; diphtheria is probably present.

I fully realize that I have made some statements in this paper, contrary to the writings of some authors; but having handled more than five thousand of these cases, am only trying to give the opinions I have formed from my observations.

